# Supplementary figures and images for: Proline-specific aminopeptidase P prevents replication-associated genome instability
Source: PLoS Genet. 2022 Jan 26;18(1):e1010025. doi: 10.1371/journal.pgen.1010025 (PMC8820600; doi:10.1371/journal.pgen.1010025)

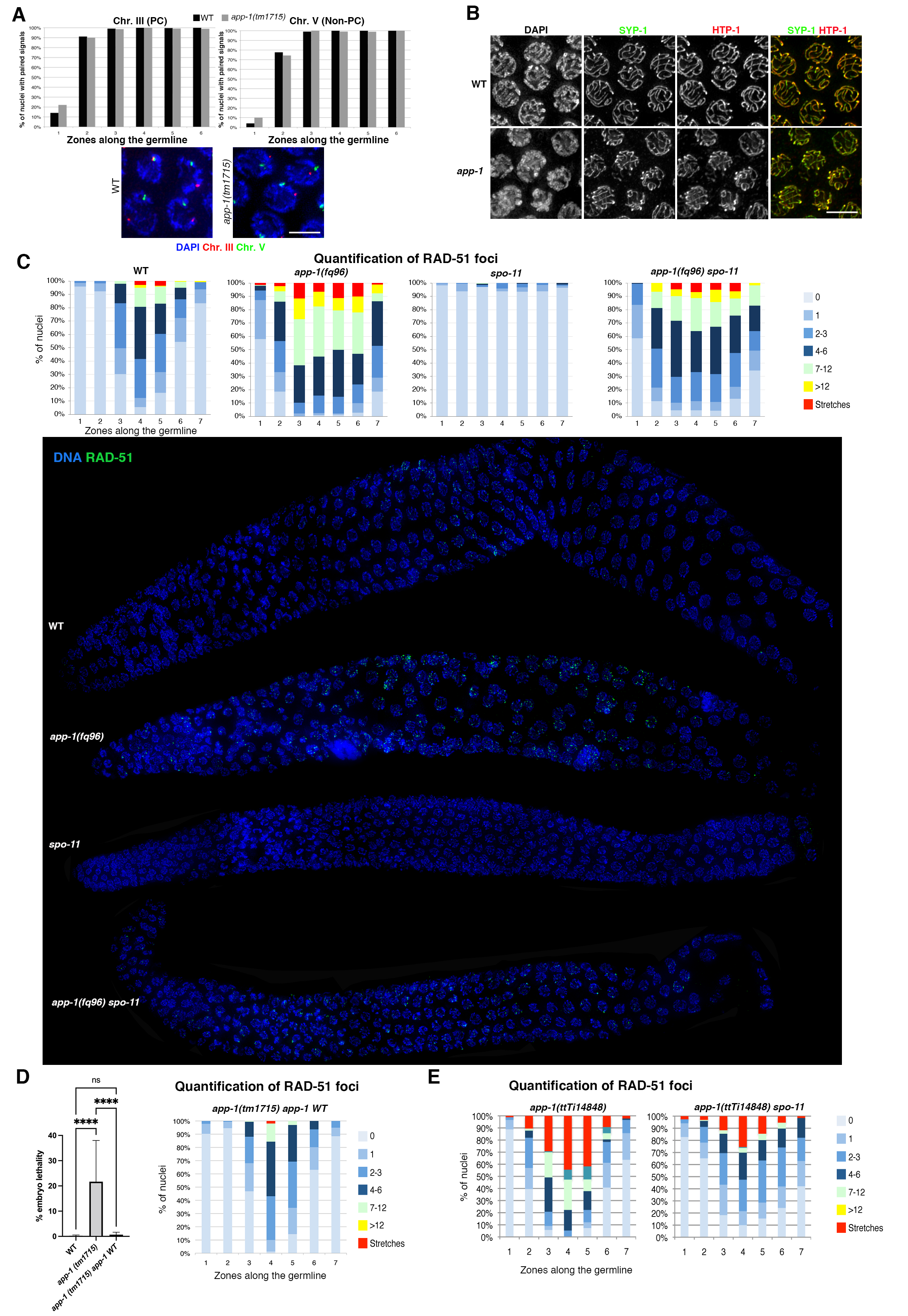

Supplement: S1 Fig — (A) (Top) Quantification of pairing levels using a probe for the pairing centre region of chromosome III (T17A3 cosmid) and a probe for 5S rDNA locus on chromosome V. Gonads were divided into six equal regions and pairing was quantified in each region. Pairing occurs normally in app-1 mutants. (Bottom) Representative images of pachytene nuclei labelled with FISH probes. The number of nuclei counted for each region were (WT, app-1): Zone 1 (77, 104), zone 2 (56, 137), zone 3 (98, 196), zone 4 (209, 173), zone 5 (198, 162), and zone 6 (89, 87). (B) Representative images of pachytene nuclei in WT and app-1 worms stained with α-SYP-1 (central component of SC) and α-HTP-1/2 (axial element components) showing normal synapsis. (C) RAD-51 foci accumulate in germ lines of app-1(fq96) and app-1(fq96); spo-11 mutants. Graphs display the regions along the germ line on the X axis (see Fig 1D for image of germ line with zones) and the percentage of nuclei with a given number of RAD-51 foci as indicated in the color key on the Y axis. The number of nuclei analysed per genotype and zone were: WT (372, 214, 144, 173, 129, 120, 111), app-1(fq96) (257, 126, 110, 97, 103, 80, 77), spo-11 (215, 180, 164, 147, 138, 102, 104), app-1(fq96); spo-11 (247, 161, 129, 132, 114, 109, 74). Examples show projections of whole germ lines of the indicated genotypes stained with anti-RAD-51 antibodies and DAPI. (D) Quantification of embryonic lethality and RAD-51 foci in worms expressing a wild-type app-1 transgene [fqSi19] in the app-1(tm1715) mutant background. Note that app-1(tm1715); fqSi19[app-1 WT] worms display low lethality and normal levels of RAD-51 foci. Number of embryos scored: WT 3148 embryos from 13 worms, app-1 (tm1715) 2073 embryos from 17 worms, and app-1(tm1715) fqSi19[app-1 WT] 4006 embryos from 19 worms and statistical significance was calculated by a one-way ANOVA test (**** P<0.0001). The number of nuclei analysed per zone for RAD-51 foci analysis were: 186, 166, 160, 160, 165, [file pgen.1010025.s001.tif]

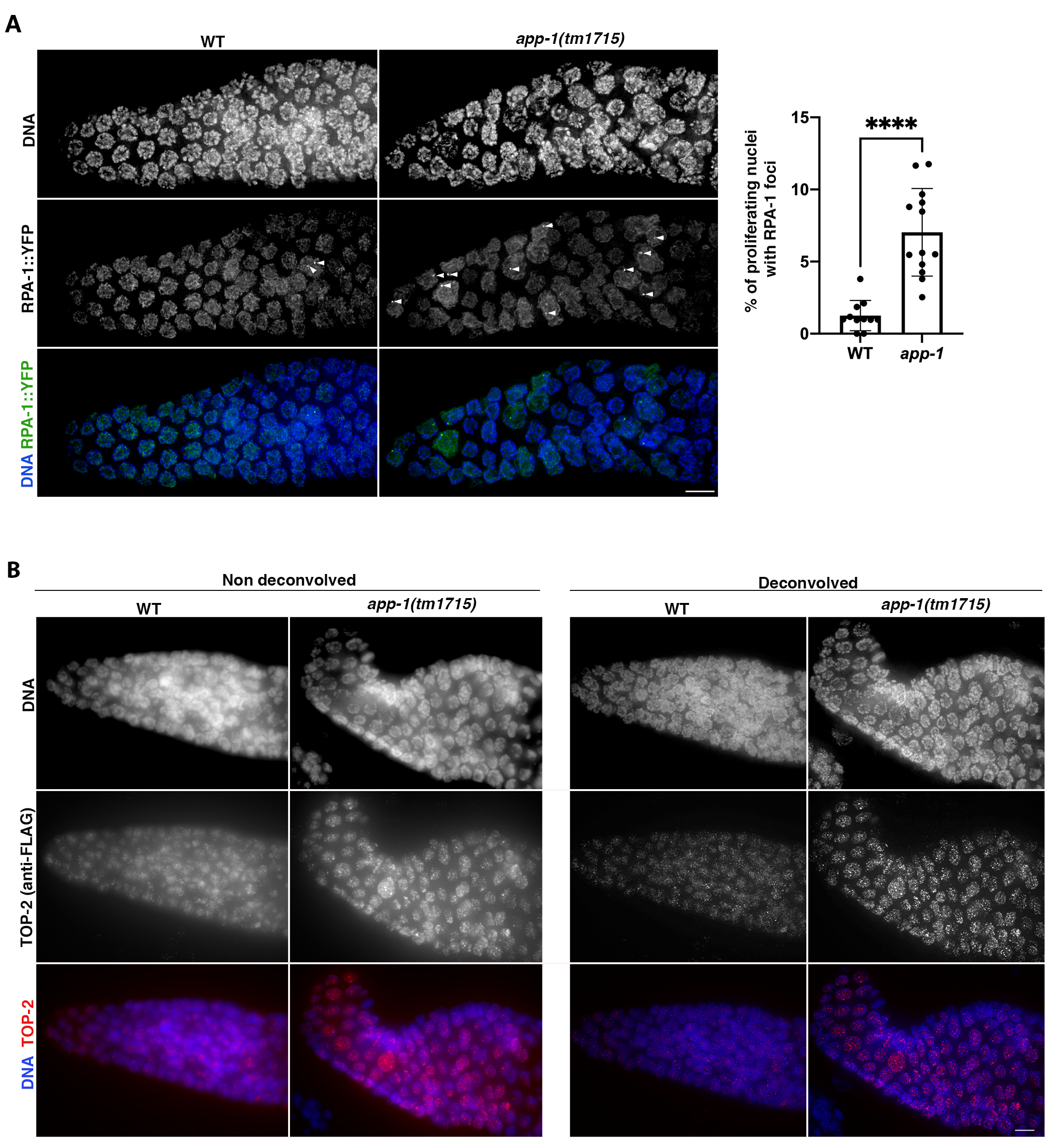

Supplement: S2 Fig — (A) Undifferentiated germ cells in app-1 mutant germlines display increased numbers of RPA-1 foci. Projections of deconvolved images from the mitotic compartment of the germ line from WT and app-1(tm1715) mutant worms carrying an rpa-1:YFP transgene. Note increased numbers of RPA::YFP foci (arrow heads) in app-1(tm1715) mutants. Graph shows % of proliferating germ-cell nuclei positive for RPA-1 per germline (10 WT and 13 app-1(tm1715) germ lines were analysed. (B) Undifferentiated germ cells in app-1 mutant germ lines display increased levels of topoisomerase II. Non-deconvolved projections of mitotic germline nuclei of worms carrying a 3XFLAG tag on the endogenous top-2 locus. Images were acquired with the same exposure and adjusted with the same settings to allow direct comparison of the intensity of TOP-2 staining (anti-FLAG antibodies) in wild-type and app-1(tm1715) mutant germ lines. Note increased levels of TOP-2 in app-1(tm1715) mutant germ line. Right-hand panels show deconvolved images of the same germ lines. Scale bar = 5 μm. See S1 Table for underlaying numerical data of graphs. (TIF) [file pgen.1010025.s002.tif]

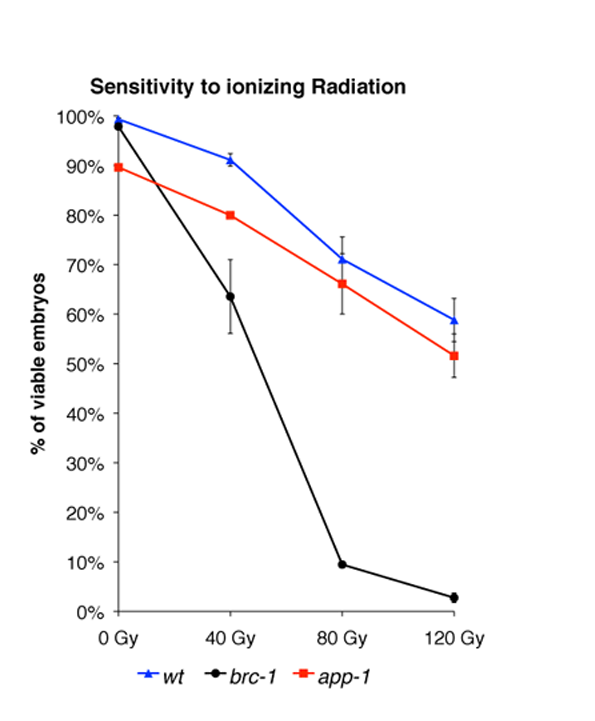

Supplement: S3 Fig — IR dose shown on X axis and viability, assessed 24 hours post irradiation, on the Y axis. app-1 mutants respond similarly to WT worms, in contrast with the hypersensitivity of brc-1 mutants used as control. Bars represent standard error of the mean (SEM). Number of embryos scored (WT, brc-1, app-1): 0 Gy (778, 753, 272), 40 Gy (643, 629, 565), 80 Gy (553, 452, 442), and 120 Gy (541, 703, 334). See S1 Table for underlaying numerical data of graphs. (TIF) [file pgen.1010025.s003.tif]

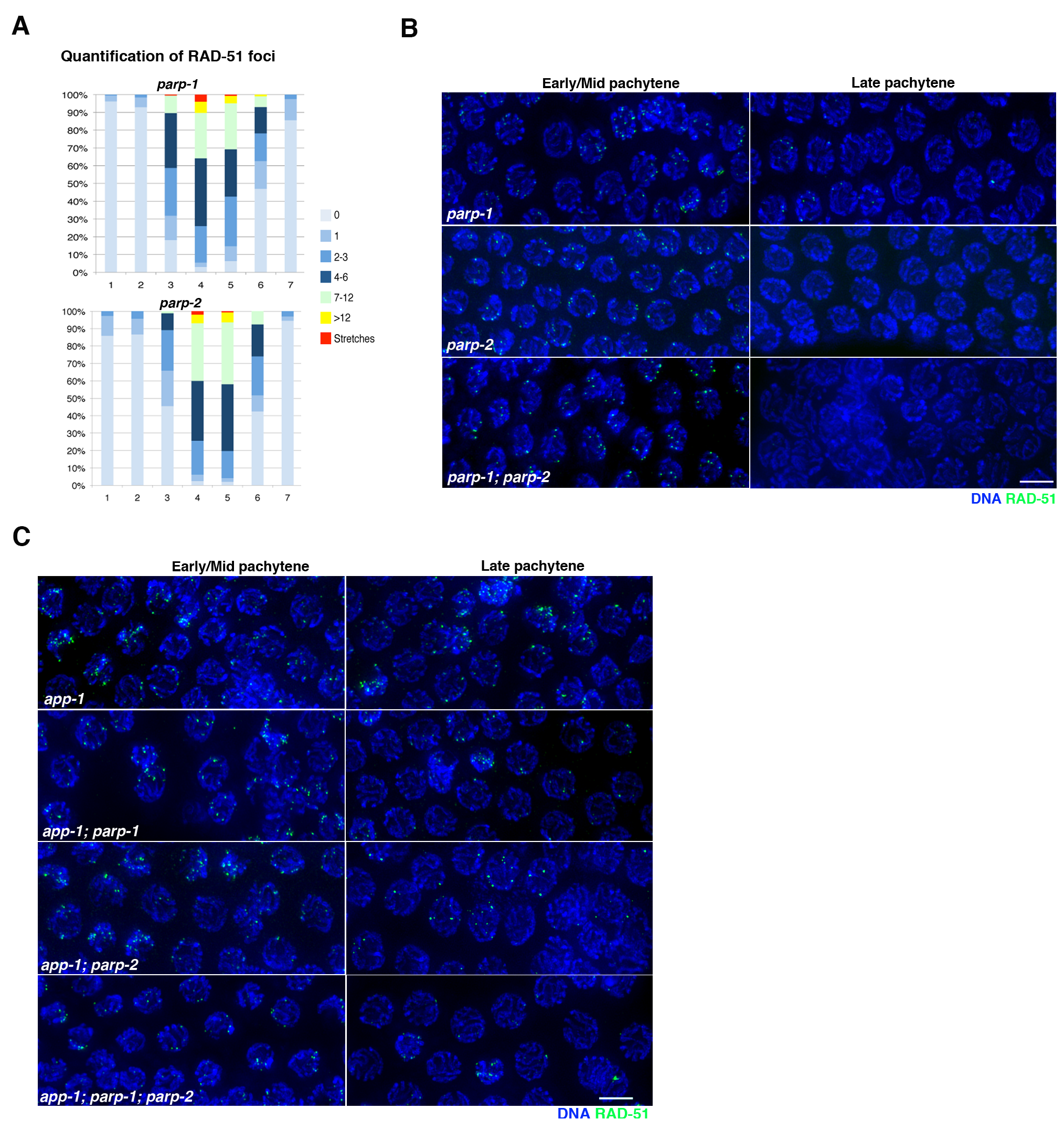

Supplement: S4 Fig — (A) Quantification of RAD-51 in germ lines of parp-1 and parp-2 single mutants show normal levels of RAD-51 foci. The number of nuclei used for RAD-51 foci quantification per genotype and zone were: parp-1 (211, 181, 182, 199, 143, 115, 160) and parp-2 (149, 210, 167, 160, 141, 174, 164). (B-C) Examples of RAD-51 staining in pachytene nuclei of indicated genotypes from graphs shown in panel A and in Fig 4B. See S1 Table for underlaying numerical data of graphs. (TIF) [file pgen.1010025.s004.tif]

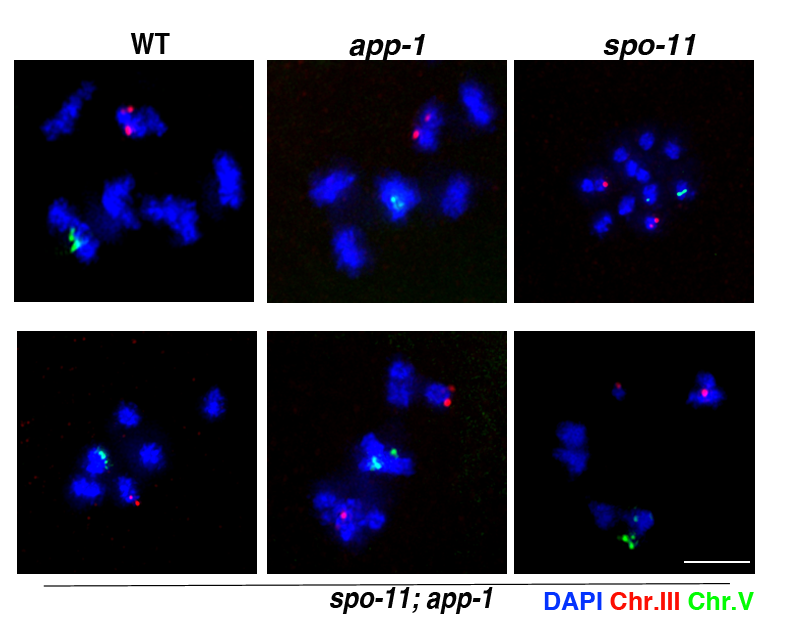

Supplement: S5 Fig — Projections of diakinesis nuclei labelled with FISH probes to visualize chromosomes III (red) and V (green). In WT and app-1 oocytes each probe is associated with a single bivalent, demonstrating attachment of homologous chromosomes by chiasmata. spo-11 oocytes lack chiasmata and therefore FISH probes label two separated chromatin bodies. Bottom panel displays three examples of oocytes from app-1; spo-11 double mutants labelled with the same FISH probes. Oocyte on the left-hand side panel displays 6 DAPI-stained bodies and each FISH probe is associated with a single chromatin mass, demonstrating attachments between homologous chromosomes. In the middle panel only four chromatin bodies are present and the probe for chromosome III is found on two different bodies, suggesting the presence of attachments between non-homologous chromosomes. The oocyte on the right-hand panel displays 5 chromatin masses; the probe for chromosome III is found in two of them, which according to size and shape may represent an isolated univalent plus a univalent fused to another unlabelled chromosome; the probe for chromosome V is found in a larger chromatin mass that also suggests the presence of attachments between non-homologous chromosomes. Scale bar = 5 μm. (TIF) [file pgen.1010025.s005.tif]

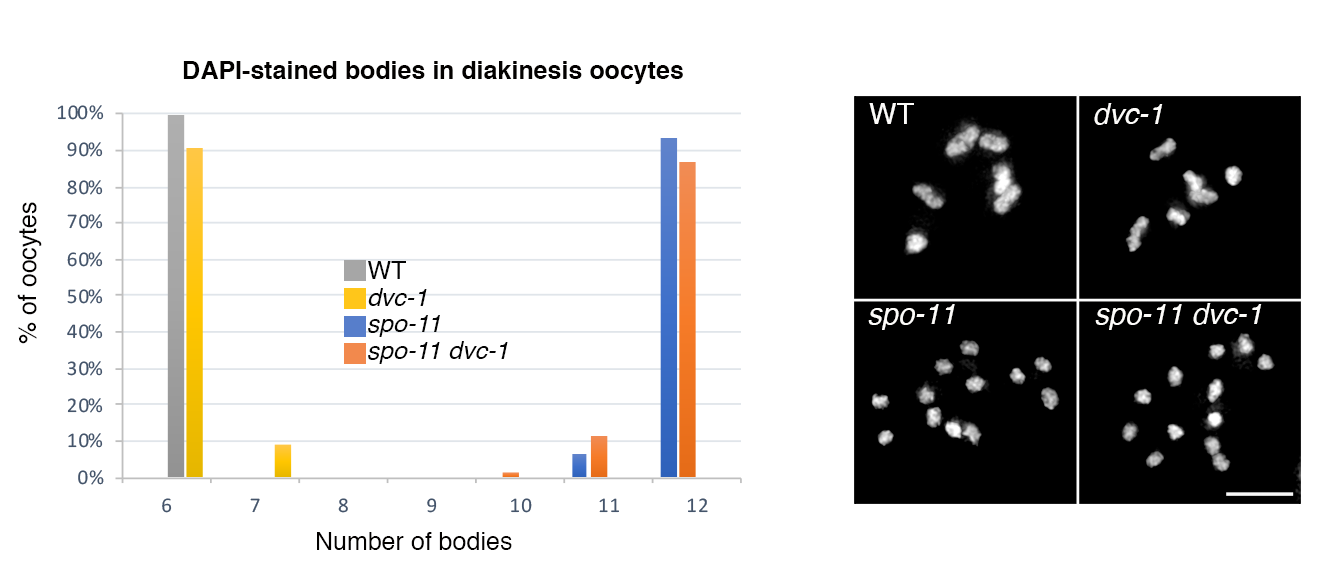

Supplement: S6 Fig — Quantification of the number of DAPI-stained bodies in diakinesis oocytes of indicated genotypes and examples shown on right-hand panel. Note that both spo-11 and spo-11 dvc-1 mutants display mostly 12 DAPI-stained bodies, indicating a failure in chiasma formation. Number of diakinesis nuclei scored: WT (20), dvc-1 (22), spo-11 dvc-1 (62), and spo-11 (45). Scale bar = 5 μm. (TIF) [file pgen.1010025.s006.tif]
