# Supplementary material for: Proline-specific aminopeptidase P prevents replication-associated genome instability
Source: PLoS Genet. 2022 Jan 26;18(1):e1010025. doi: 10.1371/journal.pgen.1010025 (PMC8820600; doi:10.1371/journal.pgen.1010025)
Supplement: Attachment — Submitted filename: Response to Reviewers comments.docx [file pgen.1010025.s008.docx]

We thank the reviewers for their overall support and constructive criticism to improve our manuscript. In response to the requests from the Editor and reviewers we have added new data showing accumulation of RPA-1 foci in undifferentiated germ cells of *app-1* mutants (Figure S2A), as well as images of RAD-51 aggregates present in *app-1* and *app-1; spo-11* mutants (Figure 1D and Figure S1C). Below we provide answers to all comments made by the three reviewers, indicating the additions and modifications that we have included in the manuscript to address them:

**Reviewer #1:** The manuscript by Silva et al. describes the unexpected finding that Proline-specific aminopeptidase APP-1, plays a role in preventing replication stress. Using genetics, cell biology and biochemical approaches the authors show convincingly that APP-1 prevents replication stress in C. elegans and extend their findings in human tissue culture. I have only minor suggestions to improve the manuscript:

1. The authors look at RAD-51 in the C. elegans germline and note that there are large aggregates of RAD-51 – mutant and wild type dont look any different to me based on the limited images shown in the supplement. Could the authors quantify this or direct the reader to what they consider aggregates?

We now provide examples of nuclei displaying RAD-51 aggregates (labelled with arrow heads) in both *app-1(tm1715)* and *app-1(tm1715); spo-11* mutants in Figure 1C. In addition, we have also added images of whole germlines from *app-1(fq96)* and *app-1(fq96); spo-11* mutants (as well as WT and *spo-11* controls) stained with anti-RAD-51 antibodies to Figure S1C.

2. In Figure 3C: please add “IR” to the figure, not just the minutes.

We have labelled the figure as requested.

3. Analysis of app-1; spo-11 double mutants suggests that the lesions produced by the app-1 mutant can serve as substrates for HR and crossover formation. Is embryonic viability improved in the app-1; spo-11 double mutant?

*app-1; spo-11* double mutants still display very high embryonic lethality. There are at least three possible explanations for this: 1) The mean number of COSA-1 foci in late pachytene nuclei of *app-1; spo-11* double mutants is 2.95, therefore not many oocytes are expected to have a full complement of 6 inter-homologue crossovers that would be required to ensure proper alignment of all homologue pairs on the first meiotic spindle. 2) The ability of a crossover to orchestrate correct chromosome segregation during the first meiotic division is largely dependent on its position along the chromosome. While the positioning of SPO-11-dependent crossovers is highly regulated to disfavour crossovers from forming in regions that will not promote correct homologue segregation (such as the center of chromosomes), it is unlikely that these crossover positioning mechanisms are fully operational over the SPO-11-independent crossovers observed in *app-1; spo-11* mutants. 3) Recovery of embryonic viability in *app-1; spo-11* double mutants would also require large rescue of crossover formation during sperm meiosis, an aspect that we have not investigated.

4. Please add statistics to the figures. Specifically, in figure 4 analyses of the genetic interaction between app-1 and parp-1/2 should be analyzed by ONE WAY ANOVA, but statistics are missing from some other figures as well.

We have calculated statistical significance using a one-way ANOVA test for embryonic lethality on Figure 4. Statistical significance for differences in the number of developmental defects in the same figure was calculated using a Chi square test as the % of developmental defects were measured by counting worms with abnormal morphology among the total hatched embryos from each genotype. We have also added statistics to the figures 1B, 1E, 6B, 7C, S1D, and S2A.

5. In the introduction on page 4, the authors state that there are “two pathways” for repair of DSBs – I recommend qualifying this to say two major/main pathways, as other pathways have been shown to play a role in the germ line.

We have added this clarification.

**Reviewer #2:** The paper by Silva at al. is well written and the conclusions are generally supported by the experimental evidence. In short, the authors find that the APP1 aminopeptidase is require for genome maintenance in the C. elegans germ line. Evidence is provided that this due to defective replication. Such defect results in increased RAD-51 focus formation, which the authors characterize reasonably well (see below). In the worm, it is known that excessive DNA double stand breaks, can bypass the need for the SPO-11 nuclease (required for meiotic double strand breaks), and this is what the authors also observe due to excessive double strand breakage in app1 mutants. They study this bypass is detail. The authors also provide evidence of increased genome instability upon APP1 depletion in mammalian cells.

In summary, in all the authors providing a solid first pass analysis of APP1 phenotypes.

We do not learn what the targets of APP1 are. This is fine, as the authors clearly acknowledging this in their discussion. All in all, it appears likely that we are looking at a DNA replication defect associated with APP1 deficiency. Experimentation, is not at the state of the art, neither in the worm nor in mammalian cells to further dissect this. As to this, in the C. elegans germ line, I think there would be a case for measuring the accumulation of RPA-1 foci, which by marking single stranded DNA are more direct indicator of DNA replication defects. Recent papers describe RPA::GFP fusions etc etc … Generally, neither in the worm, nor in U2OS cells attempts are made to study the replication defect in any detail. In the worm for instance, DNA replication defects, when associated with checkpoint activation led to prolonged embryonic cell cycles especially of the P1 cells. Specialist papers, on further dissecting replication defects have been published. Of course, there is 1001 things that could be done in mammalian cells.

Figure S2A shows increased levels of RPA-1::YFP foci in mitotically proliferating germ cells of *app-1(tm1715)* mutants.

All in all, the data is solid, this is a genetic analysis for a genetics journal. Outing myself as a geneticist, I overall support publication.

**Reviewer #3:** The manuscript of Silva et al provides detailed analysis of the role for aminopeptidase APP-1 in the C. elegans germ line showing that APP-1 prevents the accumulation of replication stress. They also show that this function of APP-1 is conserved in human cells and prevents the accumulation of nuclear-associated topoisomerase II. The data convincing show the presence of mitotic DNA damage and SPO-11 independent DNA damage. Strikingly this is the first mutant which shows such a dramatic increase in damage that can be repaired with high fidelity through the HR crossover pathway, leading to suppression of the crossover due to lack of spo-11. Overall, the data are solid and the writing is clear and the paper will be of broad interest to the DNA repair, cancer, and meiosis communities. There are minor issues with the figures and text that need to be addressed prior to publication and a few experiments that would greatly enhance this first description of the app-1 mutants. The only major issue is to clarify whether the cell cycle is simply slower or the cells arrest in response to damage since the authors propose in the discussion that APP-1 prevents the formation of replication induced damage rather than functioning in the repair.

• A description of the developmental defects quantified in Figure 1 should be provided, especially since the next paragraph states they are reminiscent of DNA damage deficient worms.

We now include a clearer description for “developmental defects” when this is first mentioned on page 7.

•. Are the brood, embryonic lethality, and developmental defects from app-1 homozygous stocks or from heterozygous mothers or which generation off a balancer? This information is not in the Methods and should be provided in the Figure legend. This is critical if app-1 is a mutator, in which case, providing info on zygotic vs maternal effect lethality should be included.

We apologise for this oversight. *app-1* mutants used for analysis reported in the paper were obtained from homozygous *app-1* mothers, not from heterozygous mothers carrying a genetic balancer. Heterozygous stocks were created for long-term storage of the strains. This information has now been added to the material and methods section.

•. Is there an increase in apoptosis in these mutants. Since the suggestion is that all the damage is repaired, one might expect there is not, this could distinguish it from other mutants and also address whether checkpoints are activated.

We have not tested whether *app-1* mutants display increased apoptosis in the germ line. Although this would be an interesting experiment, we think that it is unlikely to clarify the issue of whether all DNA damage is repaired in *app-1* mutant germlines due to the substantial presence of RAD-51 foci in zones 6-7 of the germline, where apoptotic nuclei are normally detected.

• Are the enlarged proliferative nuclei arresting in the cell cycle due to DNA damage that can be reported by activation of HUS-1-dependent checkpoint? The evidence presented is convincing that the cells are arrested and not further responded to HU because they are not cycling as quickly (though this is not explicitly stated), but the cells could be arrested in S, G2, or at the onset of M. Is there any to indicate which phase they are in? This would be a nice addition to the text.

We do not know the exact stage of the cell cycle at which proliferating germ cells arrest/slow down in *app-1* mutants, but given the morphology of enlarged nuclei (lacking evidence of chromosome condensation) we think that it is unlikely to be the onset of mitosis. To clarify if proliferating germ cells are arrested or slowed down during S-phase we have attempted to perform staining against CDC-45, which is thought to only localise to chromatin during DNA replication. Unfortunately, the staining from these experiments did not yield the resolution required to unambiguously clarify this question. Future analysis of double mutants lacking APP-1 and components of checkpoints that regulate germ cell proliferation will help clarify the point of the cell cycle at which germ cells arrest/slow down in *app-1* mutants.

• Is there also no effect on survival after HU until the higher doses?

We have not evaluated embryonic lethality following exposure to HU of adult worms. Depending on the time window between the exposure of gravid mothers to HU and the assessment of embryonic lethality this experiment would be more relevant to examine the potential contribution of APP-1 to DNA replication during early embryogenesis, an area that we have not explored on this manuscript.

•. The accumulation of TopoII in the U2OS cells could be a consequence of a cell cycle defect: increasing the number of cells in S, G2, and M relative to G1. The cells should be sorted by DNA contents and then analyzed for TopoII in G2 cells.

We agree with the reviewer that the accumulation of TopoII in U2OS cells may be a secondary consequence of the primary defect induced by APP1 knockdown. We are not suggesting that TopoII is a direct target of APP1. The goal of the experiments with U2OS cells was to test whether APP1 also contributed to genome stability in mammals, a possibility supported by the increased presence of DNA damage markers.

• Figure S2. I am not convinced by that staining shown that Topoisomerase II levels are different in the mutants because the wild type image seems “fuzzy”. Also, wouldn’t you expect the accumulation in the enlarged cells but maybe not the normal sized cells?

The non-deconvolved images shown were acquired with the same exposure and adjusted using the same settings as this provides a direct visual comparison of the overall intensity of the signal as acquired on the microscope. As noted by the reviewer, these non-deconvolved images are not as sharp as the deconvolved images, specially when the signal intensity is not very high. We now also include deconvolved images of the same germ lines showing sharper TOP-2 staining in the wild-type germ line.

• The stretches of RAD-51 that are described for app-1 mutants should be pointed to in the figure (S1C) since it appears to my eyes that the WT control also has a couple stretches, but maybe I am misinterpreting what they mean. Figure S1C should also say which “zone” the cells are from.

We have added to Figure 1 panels showing RAD-51 stretches in pachytene nuclei of *app-1* and *app-1 spo-11* mutants, using arrowheads to indicate RAD-51 signals that we considered stretches. We have also added to figure S1 examples of whole germlines from WT, *app-1*, *spo-11*, and *app-1 spo-11* stained with anti-RAD-51 antibodies

• There are a few unintuitive intellectual leaps that would help the reader better understand the phenotypes, most crticially explaining why app-1 mutants would be seemingly resistant to HU which is thought to induce replicative stress. Is it possible that APP-1 is acting through the same pathway?

We have added a sentence to the discussion (page 21) to highlight how the delayed response to HU is consistent with the presence of replication stress in *app-1* mutant germ cells. Although this observation would be consistent with APP-1 affecting the production of dNTPs, similar to HU treatment, other interpretations are also possible and therefore we prefer not to point to the regulation dNTPs as the main target of APP-1 in DNA replication.

• Figure 1C: In the image of tm1715, it appears that one of the bivalents is separating. Is this typical of the mutant?

Although we have not conducted an exhaustive examination of bivalent morphology in diakinesis oocytes of *app-1* mutants, our impression is that bivalents of *app-1* mutants look superficially normal.

•.Why are the n values given in Suppl tables except for Fig 3C, 4A? It seems these could be included in the figure legends throughout. Either way, consistency would be preferred.

We have now added all the n values to the corresponding legend and eliminated the supplemental tables displaying this information.

• In Figure S5, bottom right, the chromosome III probes appear to be on univalents based on size of the left-most DAPI spot; the right-most spot appears smaller as well and in close juxtaposition to a bivalent-like structure rather than part of it. Also, one cannot say that NHEJ is producing the observed fusions (as the title of the figure and text p 17 suggests); it could be MMEJ (or other pathways) as well.

We have reworded the text on page 17 and the legend of figure S5 to suggest that some DSBs in *app-1* mutants may be repaired by pathways other than homologous recombination, including NHEJ. We have also changed the example mentioned by the reviewer with a different oocyte showing clearer chromosome fusions.

•. Does app-1 act as a mutator? Just because there is a excessive DNA damage, if it is all repaired, it may not cause genome instability per se but rather just cell cycle delay.

Although the extensive accumulation of RAD-51 foci in the germline and the presence of worms displaying developmental defects among the progeny of *app-1* mutant worms is suggestive of some level of genome instability, we agree that we do not have sufficient data to unequivocally conclude whether the lack of APP-1 acts as a mutator. This will require for example the use of genetic reporters that monitor the appearance of mutations in specific DNA sequences, or genome sequencing across multiple generations of *app-1* mutants. These are interesting experiments, but we believe that they are outside of the scope of the current manuscript.

• Discussion: “point to APP-1 acting to prevent the onset of replication-related DNA damage rather than being required for its repair”. It might be further convincing to add that the synthetic lethality with parp1/2 support this model.

We agree with the reviewer that the synthetic lethality between *app-1* and *parp-1/2* mutants is an important finding in supporting a role for APP-1 in preventing the onset of replication-related DNA damage. As suggested, we have reiterated the synthetic lethality between app-1 and parp-1/2 mutants in this section of the discussion (page 21).

•.Discussion of the 3 models for APP-1 (at least models 1 and 2) appear vague without any analysis of whether known proteins involved in these processes might fit into these categories.

Considering that the worm proteome contains nearly 2000 potential substrates of APP-1 (proteins with proline at position 2 or 3 of their N-terminus), it is difficult to single out specific proteins as potential APP-1 targets responsible for the replication defects reported here. Moreover, the number of potential APP-1 targets is likely higher than 2000, as proteins with proline residues near their N-terminus, but beyond residue 3, could become APP-1 targets following processing of their N-terminus by other aminopeptidases. Identifying specific targets of APP-1 relevant for DNA replication is an important goal for future studies.

Textual:

We thank the reviewer for pointing out the textual issues mentioned below.

Summary: Add comma after “In this manuscript…” Done
Results line 1: Add comma after “C. elegans” Done
Fig 1E legend. Enzymatic “assay” not “essay” Done

Figure 3C, it would be helpful to indicate in the figure that this is IR (perhaps in Y axis add “post-IR) Done

Results: “The resulting app-1H392A H496A transgene (called app-1CD hereafter) was
crossed to worms homozygous for the app-1(ttTi4848) allele, which carries a
transposon insertion on the third exon of app-1 (Figure 1A) and that similar to worms
homozygous for the app-1(tm1715) and app-1(fq96) alleles also accumulate SPO-11-
independent RAD-51 foci (Figure S1F).” This is confusing. Can you say: “The resulting app-1H392A H496A transgene (called app-1CD hereafter) was tested for its ability to rescue the app-1 mutant app-1(ttTi4848) that also accumulates SPO-11-independent RAD-51 foci (Figure S1F).”
Done

Page 10, Lines 1,2: “wild-type” (with hyphen) when used as an adjective Done
Page 10, line 7. “germline” (one word) as adjective; “germ line” when noun Done

“program” not “programme” Done

p. 15 middle of the page: at times it is “mre-11; app-1” and others ”app-1; mre-11” (also com-1). Corrected to *app-1; mre-11* and *app-1; com-1*

“app-1; spo-11double mutants accumulated extensive MSH-5 and COSA-1 foci from early pachytene (Figure 6B).” This is confusing because it suggests that early pachytene is somehow different from wild type, which is not the case based on the quanitifications in the figure.

We have changed the sentence to “While MSH-5 and COSA-1 foci were largely lacking from germlines of *spo-11* mutants, *app-1; spo-11* double mutants displayed MSH-5 and COSA-1 foci from early pachytene (Figure 6B)”. We have also added statistics comparing number of MSH-5 and COSA-1 foci between *spo-11* and *app-1; spo-11* to graphs in figure 6B.

Figure 7C is discussed prior to the rest of figure 7.

We have rearranged figure 7 to solve this, old panel 7C is now 7A.

British vs American English of analyse, ionizing. Check with journal which is preferred.

We have kept British spelling for analyse and ionising.

One would expect that the mitotic damage is SPO-11-independent: “This possibility is further suggested by accumulation of SPO-11-independent RAD-51 foci in mitotic and meiotic nuclei”. Maybe should read “… accumulation of mitotic and SPO-11-independent meiotic RAD-51 foci..”

Done

P. 19 2nd line from bottom, last word should be “repair” not “repairing”

Done
